# Case 3/2016 - 58 Year-Old Hypertensive Male with End-Stage Renal Disease, Aortic Dissection, Fever and Hemoptysis

**DOI:** 10.5935/abc.20160108

**Published:** 2016-07

**Authors:** Desiderio Favarato, Paulo Sampaio Gutierrez

**Affiliations:** Instituto do Coração (InCor) HC-FMUSP, São Paulo, SP - Brazil

**Keywords:** Systemic Hypertension, End-stage Renal Failure, Aortic Dissection, Fever, Hemoptysis

The patient was a 58-year-old male, who sought medical care at InCor complaining of dyspnea and fever.

He knew he had arterial hypertension, ischemic heart disease, renal failure (undergoing renal replacement therapy) and aortic dissection. He had myocardial infarction in January 1999, being referred to InCor.

His electrocardiogram (May 31^st^, 1999) revealed: sinus rhythm; heart rate, 68 bpm; PR interval, 165 ms; QRS length, 87 ms; QT interval, 404 ms; SÂQRS -40° parallel; inferodorsal electrically inactive region (Figure 1)

**Figure 1:**
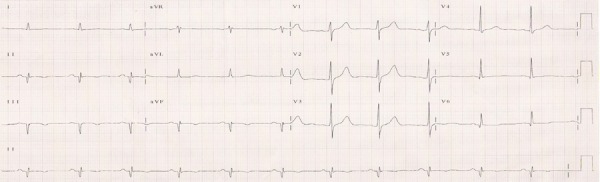
ECG Sinus rhythm, electrically inactive inferodorsal region.

His laboratory tests (May 31^st^, 1999) showed: total cholesterol, 242 mg/dL; triglycerides, 104 mg/dL; glucose, 92 mg/dL; and creatinine, 1 mg/dL.

Three years later, in 2003, the patient had encephalic vascular accident, with full recovery of force in his limbs and no sequelae.

On December 5^th^, 2005, the patient sought medical care at Hospital Mandaqui, complaining of dyspnea and chest pain.

The investigation included coronary angiography (Jan 6^th^, 2006), which revealed right coronary occlusion, 70% lesion in the middle portion of the anterior interventricular branch of the left coronary artery, 70% lesion in the first diagonal branch and irregularities in the circumflex branch. Flapping in the descending aorta was observed.

Abdominal ultrasonography (December 12^th^, 2005) showed neither liver, nor splenic or pancreatic changes. The kidneys had reduced size (right, 6x4x3 cm, and left, 7x4x3 cm), decreased corticomedullary ratio and increased echogenicity.

Renal failure was detected, and renal replacement therapy via hemodialysis was indicated.

The patient was discharged from that hospital on January 10^th^, 2006, and referred to InCor.

On medical consultation (January 14^th^, 2006), he complained of dyspnea on minimal exertion, sharp chest pain, persistent hyperthermia (38.5°C) and dry cough. His daily medication was as follows: isosorbide mononitrate (40 mg), acetylsalicylic acid (100 mg), atenolol (50 mg), amlodipine (5 mg) and simvastatin (20 mg).

On physical examination, the patient was pale, with heart rate of 63 bpm and blood pressure of 126/82 mm Hg. His examination of lungs, heart and abdomen was within the normal range. His lower limbs had decreased pulses and mild edema.

His electrocardiogram (January 13^th^, 2006) revealed: sinus rhythm; heart rate, 78 bpm; PR interval, 168 ms; QRS length, 111 ms; QT interval, 428 ms; low QRS complex voltage in the frontal plane; electrically inactive region in the inferodorsal wall (Figure 2).

**Figure 2:**
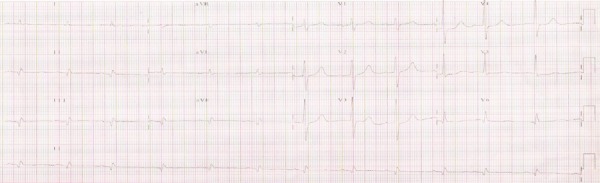
ECG (Jan. 2006). Low QRS complex voltage in the frontal plane; electrically inactive region in the inferodorsal wall.

His laboratory tests (January 14^th^, 2006) revealed: hemoglobin, 8.3 g/dL; red blood cell count, 27%; leukocytes, 10,900/mm^3^ (79% neutrophils, 2% eosinophils, 11% lymphocytes, 8% monocytes); platelets, 438,000; urea, 76 mg/dL; creatinine, 5.6 mg/dL.

His chest radiography (January 14^th^, 2006) revealed mediastinal enlargement, pulmonary opacification at the right base and marked pulmonary congestion.

Ceftriaxon was introduced to treat tracheobronchitis, and the following were maintained: amlodipine (10 mg), simvastatin (20 mg), oral atenolol (50 mg), intravenous furosemide (20 mg) and erythropoietin (4,000 IU).

Magnetic resonance imaging of the thoracic aorta (January 23^rd^, 2006) revealed aortic dissection right after the left subclavian artery emergence, extending to the abdominal aorta (Stanford type B dissection). The aorta diameter measures were as follows: root, 41 mm; ascending aorta, 34 mm; middle aortic arch, 25; descending aorta, 45 mm.

Magnetic resonance imaging of the abdominal aorta (January 23^rd^, 2006) revealed dissection from the thoracic aorta to its bifurcation (beginning of the iliac arteries), with diameter ranging from 32 mm in the suprarenal region to 22 mm in the infrarenal region. The right renal artery emerged from the false lumen, while the left renal artery emerged from the true lumen, and both were occluded. In addition, the left common iliac was occluded, being filled through collateral circulation.

The possibility of surgery was considered, but expectant management was chosen, because of type B aortic dissection and lack of lower limb ischemia.

New laboratory tests (January 23^rd^, 2006) revealed: hemoglobin, 8.3 g/dL; red blood cell count, 27%; leukocytes, 8,000/mm^3^ (71% neutrophils, 3% eosinophils, 20% lymphocytes, 6% monocytes); platelets, 483,000/mm^3^; creatinine, 4.5 mg/dL; urea, 60 mg/dL; potassium, 4 mEq/L; and sodium, 131 mEq/L.

Hemodialysis sessions were performed, and the medication maintained. The fever disappeared, and the patient was discharged from the hospital on January 26^th^, 2006.

Three and a half weeks later, the patient returned to InCor (February 19^th^, 2006) with dyspnea, fever and toxemia, in addition to passage of tarry stools suggestive of melena.

His physical examination (February 19^th^, 2006) showed paleness, tachypnea, respiratory distress, heart rate of 60 bpm and blood pressure of 120/70 mm Hg. Lung auscultation revealed no respiratory sound in the lower 2/3 of the left hemithorax and diffuse rhonchi in both hemithoraces. The heart and abdomen examinations showed no abnormalities, and the lower limbs, no edema. The peripheral pulses were symmetric.

The patient required orotracheal intubation for ventilation support. As infection of the dialysis catheter was suspected, it was withdrawn. A new dialysis catheter and double-lumen catheter were inserted, the later for the administration of intravenous drugs, such as the antibiotics vancomycin and ceftazidime.

Blood cultures (February 21^st^, 2006) grew Gram-positive cocci (*Staphylococcus aureus*), and ceftazidime was suspended.

The electrocardiogram (February 21^st^, 2006) revealed: sinus rhythm; heart rate, 73 bpm; PR interval, 167 ms; QRS length, 109 ms; QT interval, 441 ms; low QRS voltage in the frontal plane; electrically inactive region in the inferodorsal wall (Figure 3).

**Figure 3:**
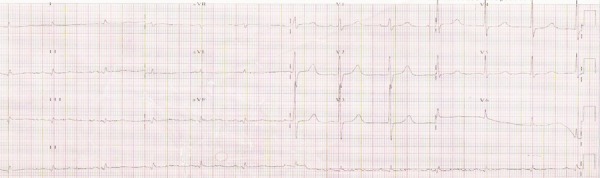
ECG (Feb. 2006). Low QRS complex voltage in the frontal plane; electrically inactive region in the inferodorsal wall.

His laboratory tests (February 21^st^, 2006) revealed: hemoglobin, 7.2 g/dL; red blood cell count, 23%; VCM, 96 µm^3^; leukocytes 11,300/mm^3^ (86% neutrophils, 11% lymphocytes, 3% monocytes); platelets, 448,000/mm^3^; urea, 126 mg/dL; creatinine, 5.6 mg/dL; sodium, 135 mEq/L; potassium, 5.4 mEq/L; arterial lactate, 17 mg/dL; ionized calcium, 1.33 mMol/L; TAP (INR), 1.42; TTPA (rel), 1.2.

Two units of red blood cell concentrate were administered (February 21^st^, 2006), increasing hemoglobin to 9 g/dL. On February 23^rd^, 2006, dialysis was performed. A new angiotomography of the aorta (February 21^st^, 2006) revealed aortic dissection right after the left subclavian artery emergence, with extensive parietal thrombi, and extending beyond the renal arteries. The left renal artery emerged from the true lumen, and the right renal artery was not observed (thrombosed).

His laboratory reassessment (February 24^th^, 2006) revealed: hemoglobin, 9.1 g/dL; red blood cell count, 28%; leukocytes, 9,300/mm^3^ (86% neutrophils, 2% eosinophils, 6% lymphocytes, 6% monocytes); platelets, 240,000/mm^3^; urea, 85 mg/dL; creatinine, 0.6 mg/dL; sodium, 133 mEq/L; potassium, 3.9 mEq/L; ionized calcium, 1.29 mMol/L.

The patient had back pain and massive hemoptysis, with cardiopulmonary arrest in asystole, did not respond to resuscitation maneuvers, and died (February 24^th^, 2006).

## Clinical aspects

The patient had a previous diagnosis of arterial hypertension and chronic renal disease, with renal failure, being on renal replacement therapy via hemodialysis. He had chest pain and dyspnea. His clinical and laboratory assessments revealed coronary artery disease and chronic aortic dissection.

Chronic renal disease significantly progresses as age and cardiovascular disease advance, culminating with renal function worsening. The ARIC Study reports an increase in cardiovascular disease prevalence from 18% to 40% before the age of 65 years, from normal renal function to a glomerular filtration rate drop of 15 mL/min/1.73m^2^, while, over the age of 65 years, that prevalence increases from 20% to 50%, with progressive renal function worsening.^1^

In addition, with renal function deterioration, mortality due to cardiovascular disease increases, from 27.5% to 58%.^2,3^

Specifically in individuals undergoing dialysis, cardiovascular causes account for 36% of the deaths in the first six months of that therapy. In addition, in that subgroup of patients dying from cardiovascular disease, 64% do it suddenly, 16% die from heart failure, 10%, from acute myocardial infarction, 6%, from encephalic vascular accident, and 4%, from other causes. After six months of dialysis, cardiovascular diseases account for 44.1% of the deaths, maintaining the proportion between the cardiovascular causes.^4^

That association between atherosclerosis and renal disease is not random, resulting rather from the fact that those diseases share the same risk factors, with acceleration of atherosclerosis due to changes in the calcium and homocysteine metabolism in renal failure.^5^

Those facts make coronary artery disease frequent in those patients, reaching almost 60% of the cases undergoing assessment for renal transplantation.^6^

The pain of the patient in question might not have resulted from occlusion of the right coronary artery, because he already had myocardial infarction in the inferior wall seven years before the final event.

The patient's dry cough and fever were attributed to an episode of pneumonia, supported by the finding of pulmonary opacification at the right base. However, hyperthermia not rarely accompanies aortic dissection, affecting, in some case series, up to 30% of the patients; therefore, it should be included in the differential diagnosis of aortic valve endocarditis.^7,8^

Usually, Stanford type B aortic dissection does not require surgery, except in the presence of ischemia of organs or limbs, because mortality of Stanford type B aortic dissection is far lower than that of ascending aorta dissection (close to 90% in the acute phase). In the case series by Parsa et al.^9^, patients with type B aortic dissection without persistent chest pain or hypertension had mortality of 4%, and those with chest pain or hypertension, of 17%.

The expectant management with drug treatment for Stanford type B aortic dissection adopted in the present case is in accordance with the recommendations of international guidelines for such cases.

The endovascular treatment, which does not result in a mortality reduction, has potential complications inherent in the procedure, such as encephalic vascular accident, paraparesis and death, and those resulting from stent implantation, such as retrograde dissection and stent leak.^10-12^

In the Internacional Registry of Acute Aortic Dissection (IRAD), 24% of type B dissections underwent emergency surgery in the first two weeks due to complications, such as poor perfusion, hemorrhagic pleural effusion, periaortic hematoma, refractory pain and hypertension.^13^

The complications of the chronic phase of type B dissection are: aneurysmal dilation over 5.5 cm; 4-mm annual increase in aortic diameter; symptom recurrence despite optimal drug therapy.^14^

In that same IRAD, 31% to 66% of the deaths after discharge were associated with dissection.^13^ (**Desiderio Favarato, MD**)

**Diagnostic hypothesis:** Aortic dissection with rupture to the pleural cavity, and sepsis due to infection related to venous access (positive blood cultures for *Staphylococcus aureus*). (**Desiderio Favarato, MD**)

## Postmortem examination

**Figure 4:**
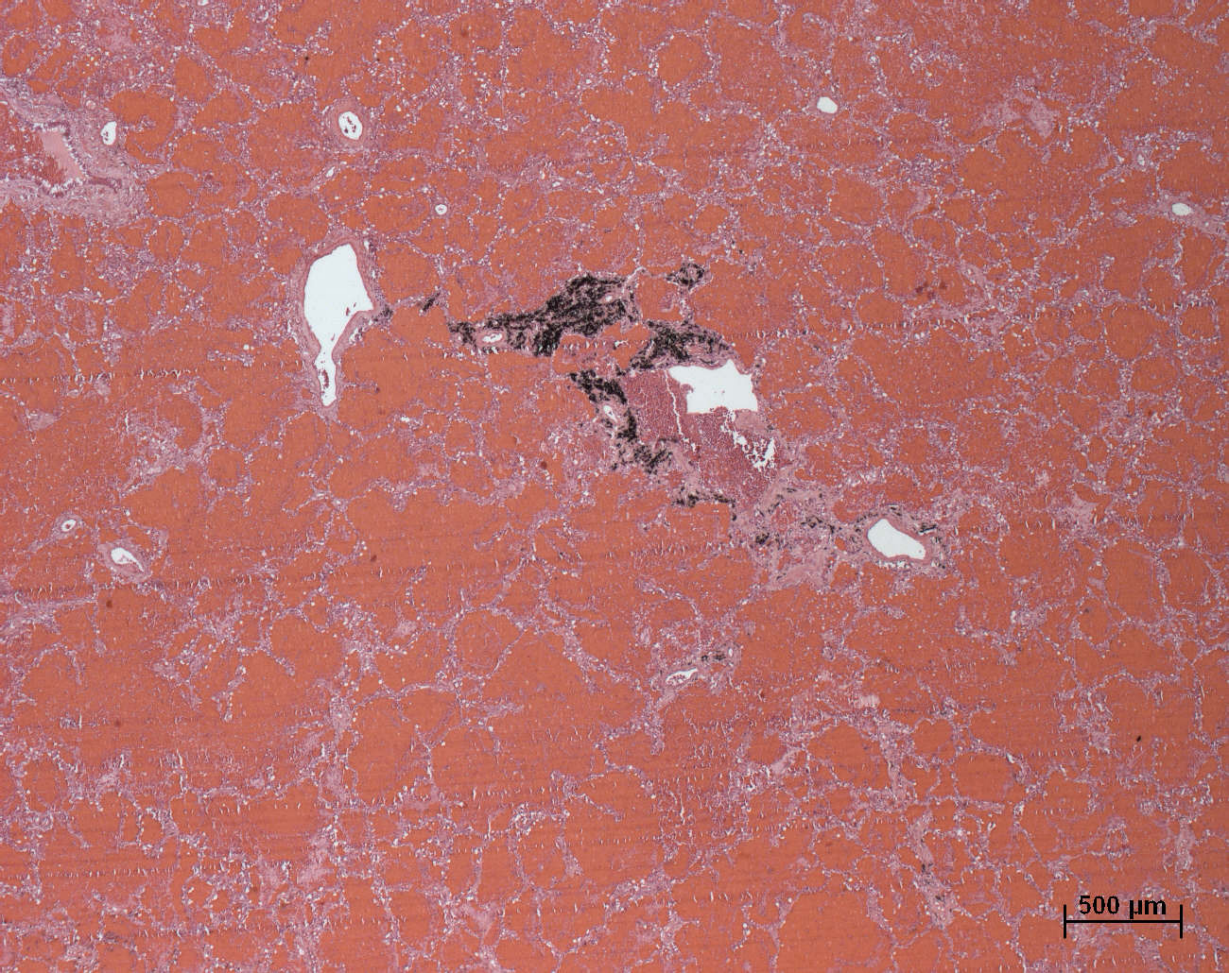
Microscopic section of the left lung showing massive pulmonary hemorrhage, with alveolar spaces filled with red blood cells. Note the small area with anthracotic pigment. Hematoxylin-Eosin stain, x2.5.

The patient's main disease was chronic aortic dissection, DeBakey type III (Stanford type B), extending from the aortic arch to the iliac bifurcation. The entrance orifice had 2 cm of extension. There was aortic rupture to the left lung and pleural cavity, with massive pulmonary hemorrhage (Figure 4), which was the final factor triggering death.

In association with dissection, the patient had systemic arterial hypertension, morphologically represented by benign nephrosclerosis and concentric left ventricular hypertrophy.

**Figure 5:**
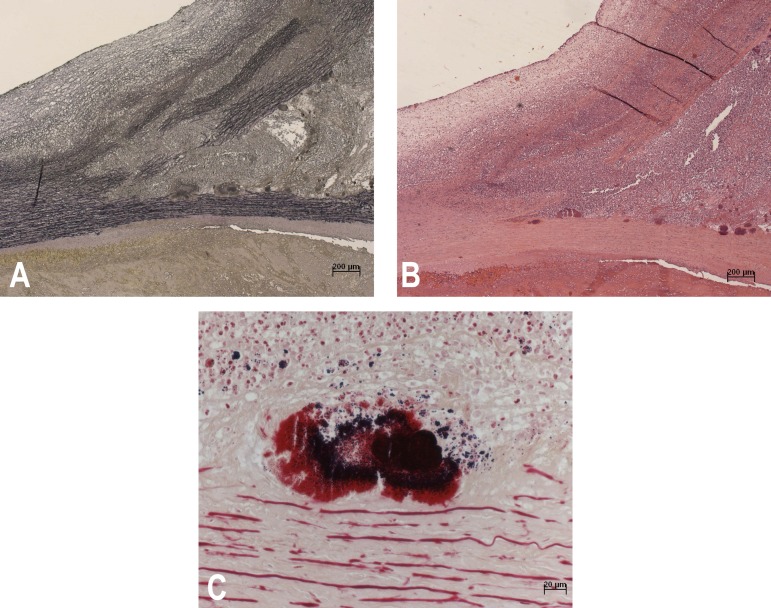
A: Microscopic section of the aorta, with elastic layers stained in black. Note their fragmentation and cleavage, characterizing aortic dissection. (Verhoeff stain, x10). B: Same aortic region showing intense inflammatory infiltrate with predominance of polymorphonuclear neutrophils. (Hematoxylin-Eosin stain, x10). C: Same aortic region showing colony of Gram-positive cocci. (Brown and Hopps stain, x10).

The microscopic study of the aorta evidenced aspects commonly related to aortic dissections, such as wall delamination of tunica media (Figure 5A) and areas with mucoid material accumulation, in addition to an intense acute inflammatory process, with a large number of polymorphonuclear neutrophils (Figure 5B) and numerous bacterial colonies of Gram-positive cocci (Figure 5C). The other organs showed no infection.

In addition to aortic dissection, the patient had atherosclerosis, affecting the abdominal aorta, the cerebral territory, with old infarctions in the cerebellum and right temporal lobe, and the coronary arteries, with healed infarction in the left ventricular posterior wall (inferior, diaphragmatic). In addition, there was superficial chronic gastritis, with hemorrhagic gastric content. (**Paulo Sampaio Gutierrez, MD**)

**Anatomopathological diagnosis:** systemic arterial hypertension and chronic aortic dissection, DeBakey type III, with bacterial infection caused by Gram-positive cocci in the false lumen wall.

**Cause of death:** massive pulmonary hemorrhage due to aortic rupture. (**Paulo Sampaio Gutierrez, MD**)

## Comments

Neither the patient's disease - aortic dissection in a hypertensive patient - nor his cause of death - aortic rupture to the lung - is uncommon. The patient had a period of relative stability; because the dissection was restricted to the descending aorta, and there was no lower limb ischemia, expectant management was adopted. Aortic rupture, however, was unexpected.

The postmortem examination evidenced an uncommon complication to be the cause of aortic rupture: bacterial colonization of the aortic wall, with numerous colonies of Gram-positive cocci and positive blood cultures for *Staphylococcus aureus*. That infection triggered intense acute inflammatory reaction, with inflammatory cells releasing lytic substances, which leads to tissue disintegration, including the extracellular matrix, and eventually caused aortic rupture.

The contamination of the false lumen with infectious agents is rare: PubMed system shows only ten cases, some of which in patients with neoplasms.^15-19^ (**Paulo Sampaio Gutierrez, MD**)

**Section editor:** Alfredo José Mansur (ajmansur@incor.usp.br)

**Associated editors:** Desidério Favarato (dclfavarato@incor.usp.br)

Vera Demarchi Aiello (anpvera@incor.usp.br)
